# Development of geographic inequality in dental caries and its association with socioeconomic factors over an 18-year period in Denmark

**DOI:** 10.1186/s12903-023-03373-5

**Published:** 2023-09-14

**Authors:** Kaushik Sengupta, Kristine Bihrmann, Lisa Bøge Christensen, Laust Hvas Mortensen, Ingelise Andersen, Annette Kjær Ersbøll

**Affiliations:** 1 Section of Social Medicine, Department of Public Health, Faculty of Health and Medical Sciences, University of Copenhagen, Copenhagen, Denmark; 2 National Institute of Public Health, University of Southern Denmark, Copenhagen, Denmark; 3 Department of Odontology, Faculty of Health and Medical Sciences, University of Copenhagen, Copenhagen, Denmark; 4 Methods and Analysis, Statistics Denmark, Copenhagen, Denmark

**Keywords:** Social class, Adolescent, Health status disparities, Spatial analysis, Trends

## Abstract

**Background:**

Few studies have examined the development of geographic and socioeconomic inequalities in caries over time or have simultaneously assessed individual-level socioeconomic position (SEP) and neighborhood-level factors as a multi-layered phenomenon influencing caries inequalities. This study examined (i) the trends in geographic inequalities in caries among adolescents in Denmark and (ii) how the association between SEP and caries has progressed over time, when accounting for individual and neighborhood-level confounding factors.

**Methods:**

This nationwide repeated cross-sectional study included 15-year-olds in Denmark from 1995, 2003, and 2013 (*n* = 149,808). The outcome was caries experience (measured by the decayed, missing, and filled tooth surfaces [DMFS] index). The exposure of interest was SEP, indicated by the previous year’s parental education, occupational social class, and (equivalized) disposable household income. Covariates included individual-level factors (immigration status, country of origin, number of children and persons in the family, and household type) and neighborhood (residence municipality)-level factors (Gini index; proportion of unemployed, low-educated, and unmarried/non-cohabiting individuals; proportion of single-parent households and households with overcrowding). Data sources included the Danish national dental and administrative social registers and Statistics Denmark’s statistics database (StatBank). Data were analyzed using spatial and spatiotemporal modelling utilizing zero-inflated negative binomial regressions and integrated nested Laplace approximations for Bayesian parametric inference. Observed caries experience geo-maps of the Danish municipalities for 1995, 2003, and 2013 were created.

**Results:**

Between 1995 and 2013, caries prevalence in the 15-year-olds declined sharply (1995, 71%; 2013, 45%). Caries experience declined in nearly all socioeconomic subgroups and municipalities. However, geographic inequalities persisted with higher caries levels largely concentrated in the relatively deprived areas of Denmark. Increasing relative socioeconomic inequalities in caries over time were observed with significant graded associations between SEP and caries despite adjustment for the various individual and neighborhood-level covariates and the effect of assessment year (e.g., 15-year-olds with parents having basic education had 1.91-fold [95% CI: 1.86–1.95] higher caries experience than those having parents with high education).

**Conclusions:**

Reducing these enduring inequalities will likely require additional resources and targeted supportive and preventive measures for adolescents from lower SEP backgrounds and those residing in municipalities with higher caries prevalence.

**Supplementary Information:**

The online version contains supplementary material available at 10.1186/s12903-023-03373-5.

## Introduction

Dental caries is one of the most common chronic diseases in humans. Data from the Global Burden of Disease Study 2019 indicate that caries in permanent teeth is the most common disease globally, affecting an estimated 2.3 billion people [1]. A high prevalence of caries is seen even in Scandinavian countries, which provide universal access to dental care for children and adolescents; for example, a recent Danish study showed that caries in permanent teeth still affects approximately 50% of the adolescent population [2]. Existing Danish studies have also indicated socioeconomic position (SEP) to be a key determinant of dental caries experience in the adolescent population; consequently, those from relatively more socioeconomically disadvantaged backgrounds tend to experience relatively higher levels of caries [2, 3].

Apart from SEP and other individual-level determinants, inequalities in caries experience may be shaped by the neighbourhood (or geographical context) in which an individual grows up. Neighbourhood-level caries determinants include both compositional and contextual factors. Compositional factors are the demographic and socioeconomic attributes of the individuals living in a neighbourhood [4]. Contextual factors, on the other hand, are the intrinsic characteristics of the neighbourhoods themselves, above and beyond those of the residing individuals (e.g., dental and healthcare system organisation, community norms and resources, laws and regulations) [5]. Studies examining the association between neighbourhood socioeconomic conditions and caries have found marked socioeconomic inequalities in caries experience among adolescents at the neighbourhood level, even in countries with universal care policies, such as Denmark and Sweden [6–8]. Nevertheless, most of the existing studies have not examined the development of these inequalities over time. Moreover, few studies have simultaneously assessed individual-level SEP and neighbourhood-level factors as a multi-layered phenomenon influencing caries inequalities. The effect of SEP on caries cannot be fully understood except in the context of neighbourhood-level influences.

Denmark is a country of approximately 5.9 million individuals, of whom approximately 85% are of Danish origin with the remaining 15% being immigrants and descendants of immigrants from Western and non-Western countries. The growth rate is around 1%, and the fertility rate is 1.55. The age group of 0 to 14 years covers almost 17% and the age group of ≥ 65 years, around 20%. Prior to 2007, Denmark’s administrative structure comprised 275 municipalities; a structural reform in 2007 resulted in a changed local and regional administrative structure with 98 municipalities (several smaller municipalities were consolidated into larger units) and 5 regions. The municipalities are responsible for a number of primary health and social services, for example, child dental treatment, child nursing, elderly care services, rehabilitation outside hospital, home nursing, and physiotherapy. In addition, municipalities co-finance regional rehabilitation services and training facilities. Denmark has a long history of collecting and storing social- and health-related information on the entire population using national registries; this includes historical and contemporary information on individual and neighbourhood socioeconomic characteristics. This provides a unique opportunity to gain a more detailed insight into the role of individual-level characteristics, neighbourhood-level influences, and time in shaping inequalities in caries. In this study, such detailed multi-level data from 15-year-olds from three different years, namely, 1995, 2003, and 2013, were used. One objective was to examine the trends in geographic inequalities in caries among adolescents in Denmark, with geographic neighbourhoods defined as residential municipalities. Another objective was to assess how the association between SEP and caries in adolescents have developed over the years when adjusted for various potential individual- and neighbourhood-level confounders. Such an expanded multi-level focus on caries determinants should contribute to a more comprehensive understanding of the drivers behind dental caries inequalities.

## Methods

### Study design and population

This was a nationwide, register-based repeated cross-sectional study. It was conducted on all individuals in Denmark born in 1980, 1988, and 1998, who consequently became 15 years old in 1995, 2003, and 2013, respectively. The age group of 15 years was chosen as it is a World Health Organization (WHO)-recommended index age group for conducting oral health assessments in children [9].

### Data sources

Dental caries information was compiled from the national dental database, Sundhedsstyrelsens Centrale Odontologiske Register (SCOR). SCOR has been administered since 1972 by the Danish Health Authority. In SCOR, dental professionals systematically record data from dental visits made by children and adolescents participating in the national dental service. The register covers about three-fourths of all Danes < 18 years of age, particularly the age groups of 5, 7, 12, and 15 years who are expected to undergo a mandatory dental examination [7]. At the time of conduct of the study, the most recent and updated information in SCOR was available for 2013 while the earliest good quality data were obtainable for 1995.

The registers used to obtain sociodemographic data in this study included the population [10], education [11], income and transfer payments [12], labour market affiliation [13], and household and family registers [14]. A unique personal identification number (PIN) is assigned to each resident in Denmark at birth or immigration and is managed by the Danish Civil Registration System (CRS) [15]. This PIN was used to identify, extract, and link each 15-year-old’s data from the national registers with clinical data from SCOR. The Danish CRS includes PINs of parents, which were used to obtain information on parental social variables. Data on neighbourhood characteristics were computed from information publicly available at Statistics Denmark’s Statbank [16].

### Study variables

#### Outcome

The outcome of interest was dental caries experience in permanent teeth, except third molars. This was measured for each adolescent using the DMFS index. This index is a count of the number of decayed (D), missing (M), and filled (F) tooth surfaces. It represents the cumulative amount of dental caries experience in an individual [9]. In this study, for the D component, only cavitated lesions (i.e., manifest caries) were considered.

#### Exposures

SEP for the 15-year-olds was indicated by parental education, income, and occupational social class. The 15-year-olds were categorised into parental educational subgroups based on the highest attained educational level between the parents, as recorded in the previous year (i.e., 1994, 2002, and 2012). These subgroups were classified as (i) basic (education duration, ≤ 10 years; level, lower secondary or less), (ii) medium (11–12 years; upper or post-secondary), and (iii) high (≥ 13 years; tertiary education) according to the 2011 International Standard Classification of Education levels 0–2, 3–5, and 6–8, respectively [17]. The 15-year-olds were also assigned to one of four household income quartiles. For this, information on the previous year’s equivalised disposable household income was obtained from the registries and categorised into quartiles. Disposable household income is equivalised in the registries according to the Organisation for Economic Co-operation and Development’s (OECD) modified scale [18].

Family occupational social class was coded according to the Danish Occupational Social Class classification into four groups: (i) high (occupations requiring the highest level of skills, e.g., executives in companies, self-employed individuals with ≥ 5 employees), (ii) intermediate (occupations requiring non-manual, intermediate-level skills, including self-employed individuals with < 5 employees), (iii) basic (occupations necessitating basic or manual-level skills [adolescents whose parents are currently pursuing further education were included in this group]), and (iv) out of labour market (currently unemployed parents, including those receiving social benefits). This was based on the highest recorded occupation between the two parents in the previous year.

#### Confounders

##### Individual-level variables

Individual-level confounders included information on (i) country of origin (Western [Nordic and Western European countries, USA, Canada, Australia, and New Zealand] and non-Western countries), (ii) immigration status (immigrants [those born outside Denmark to parents who have either foreign citizenship or were themselves born outside Denmark], descendants of immigrants [those born in Denmark to parents who are both immigrants or descendants and are not Danish citizens], and ethnic Danes [those born in Denmark and having at least one parent with Danish citizenship]), (iii) household type (living with both parents [traditional family], one parent having a new partner [reconstructed family], a single parent [single-parent family], or not living with parents), (iv) number of persons in the family (1–2, 3–5, and > 5), and (v) number of children in the family (1, 2, 3, and ≥ 4).

##### Neighbourhood-level variables

Each 15-year-old was geo-coded with respect to his/her residence municipality. To determine the residence municipality, we used the pre-2007 municipality structure in Denmark (which comprised 275 administratively defined municipal areas). Several indicators of socioeconomic deprivation for the 275 former municipalities were obtained from Statistics Denmark for each study year; these included the proportion of (i) unemployed, (ii) low educated (basic education), (iii) unmarried/non-cohabiting individuals, (iv) households with overcrowding (person per room ratio > 1), (v) non-home ownership, and (vi) single-parent households [16]. Moreover, (vii) the Gini index for each municipality in each of the three years was obtained from Statistics Denmark.

### Data management, statistical analysis, and geo-mapping

Adolescents were excluded from the analyses if they lacked dental data in SCOR or had missing information on gender, residence municipality, or one of the SEP variables. Those with negative household income were also excluded to avoid misclassification of income, as they could either be from poor families or rich business households registering negative income in a particular year.

Data management and statistical analyses were accomplished using SAS (version 9.4) and R (version 3.5.0). Individual- and neighbourhood-level data were described using frequency distributions, or prevalence proportions. The associations between the SEP variables and caries were analysed using Bayesian zero-inflated negative binomial regression models. A zero-inflated model was chosen to account for the high proportion of DMFS counts being 0 [2]. The three individual-level SEP variables were included individually as fixed effects; the models were adjusted for individual- and neighbourhood-level confounders (as fixed effects). The models also included a random effect component, modelled using a Besag-York-Mollié (BYM) model [19]; this component included (i) a geographically (spatially) structured term (to account for any spatial autocorrelation of DMFS values) and (ii) an unstructured term to account for any overdispersion of the data. Spatial autocorrelation, i.e., clustering of DMFS values in neighbouring municipalities, is possible as in general, neighbouring geographic areas tend to have similar health outcomes than those farther apart [20]. Spatial autocorrelation was accounted for by creating a binary neighbourhood adjacency matrix representing the adjacency structure of the Danish municipalities (a 275 × 275 matrix in which municipalities that shared a common boundary were coded as neighbours; the Danish islands were coded as neighbours to the municipalities to which they were connected by ferry or bridge). The geographically structured random effect was assigned an intrinsic conditionally autoregressive (CAR) prior, the hyperparameter of which was assigned a log-gamma distribution with parameters (1, 0.001). The unstructured random effect was modelled assuming an exchangeable Gaussian prior [21]. The fixed effects were assigned default Gaussian (0, 0.001^−1^) prior distributions.

Using these zero-inflated BYM CAR models, two separate analyses were performed. The first analysis was a spatial analysis, stratified by study year (Model 1). The second analysis was a spatiotemporal analysis in which data from the three study years were combined and a joint analysis performed with “year” additionally included as a fixed effect (Model 2). For both analyses, integrated nested Laplace approximations (INLA) in R were used for Bayesian parametric inference [22]. Parameter estimates of the fixed-effect variables were derived using posterior mean DMFS ratios (i.e., ratios of mean DMFS scores from the various SEP and other fixed-effect variable subgroups), while the 2.5 and 97.5 percentiles were used to define the corresponding 95% credible intervals (CIs). For each variable, the subgroup having the lowest mean DMFS score (i.e., the best-off category) was used as the reference for obtaining the ratios. The relative percentage contribution of the geographically structured random effect to the total variance was calculated using a simulation-based approach as described elsewhere [21].

Subsequently, one sensitivity analysis was performed to evaluate the impact of the chosen prior distributions on the parameter estimates. This was assessed by changing the parameters of the specified distributions to the default values supplied by INLA (Model 3).

#### Caries experience geo-maps

To visually examine the changes in the geographic patterning of caries across the study period, we created observed caries experience geo-maps of the Danish municipalities for 1995, 2003, and 2013. The observed mean DMFS values were mapped for each municipality in each year. The mean DMFS values were categorised as 0.0–1.9, 2.0–3.9, 4.0–5.9, 6.0–7.9, and ≥ 8.0, which correspond to the categories used by the Danish Health Authority in its annual dental health reports [23].

## Results

**Fig. 1:**
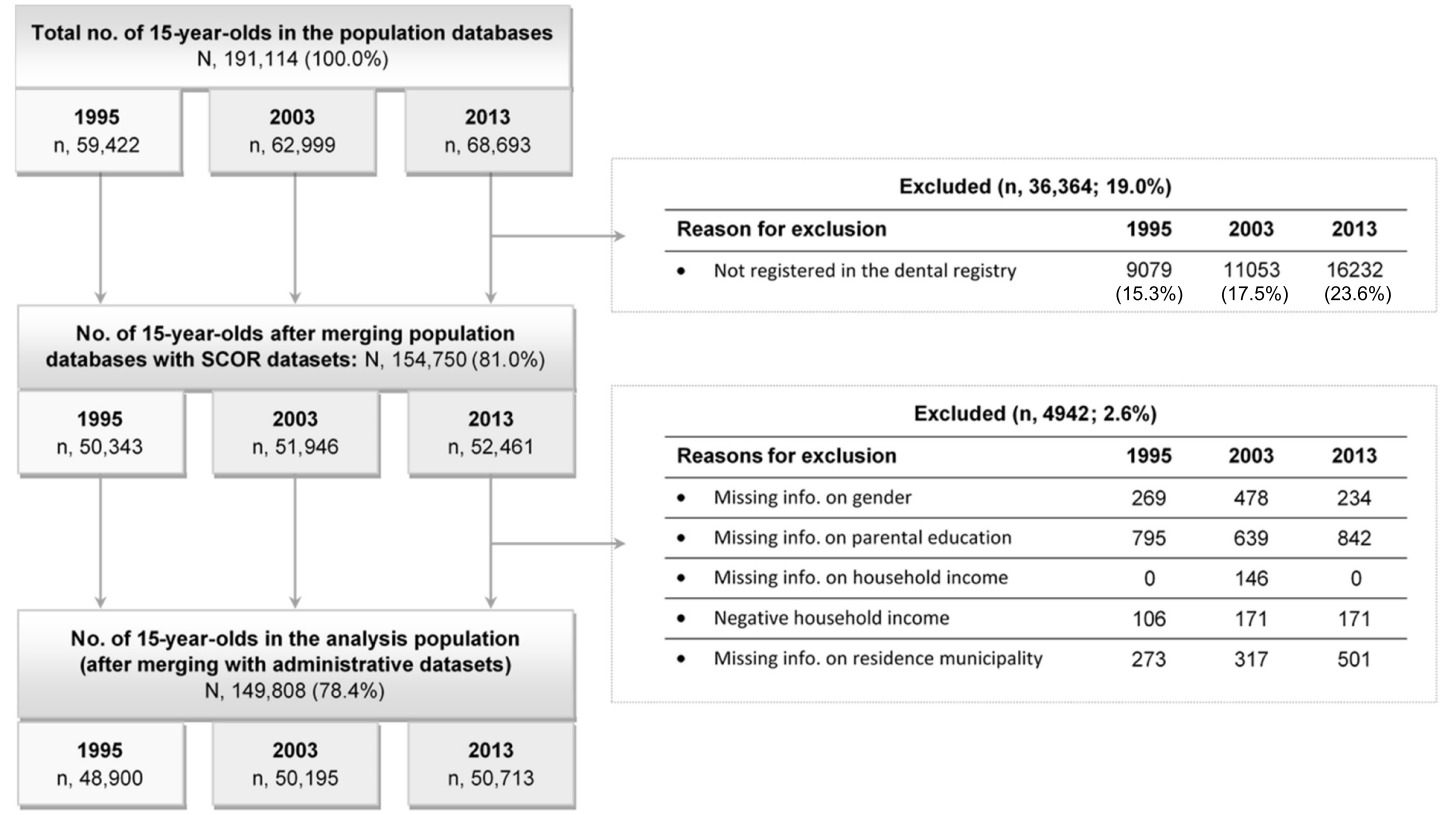
Flow diagram showing the number of 15-year-olds included in the analysis in 1995, 2003, and 2013

In 1995, 2003, and 2013, respectively, 15.3%, 17.5%, and 23.6% of the adolescents did not have any dental data in SCOR and were excluded (Fig. 1: Flow diagram showing the number of 15-year-olds included in the analysis in 1995, 2003, and 2013). When these individuals were compared with those having dental data in SCOR, no marked differences in the distribution of the sociodemographic variables were observed (Appendix 1 (a) 1995, (b) 2003, (c) 2013). In the three study years, among those with dental data, ≤ 0.3% had negative household income and approximately 2% had missing information on residence municipality or one of the SEP variables; these individuals were also excluded. Thus, from a nationwide population of all 15-year-olds in Denmark in 1995, 2003, and 2013, 82.3% (*n* = 48,900), 79.7% (*n* = 50,195), and 73.8% (*n* = 50,713), respectively, were included (approximately 97% of all adolescents with dental data in SCOR in each year) (Fig. 1).

The prevalence of dental caries experience (DMFS > 0) in the overall study population was 71% in 1995, which declined to 63% in 2003 and 45% in 2013 (data not shown in tables). Similarly, the average caries experience declined over the years, with the mean DMFS (5th–95th percentile) in the overall study population reducing from 4.11 (0–15) in 1995 to 3.11 (0–12) in 2003 and 1.81 (0–8) in 2013 (not shown in tables). Mean DMFS also declined over time in almost all socioeconomic subgroups and in 260 (94.5%) Danish municipalities. In total, 25 municipalities had over 75% reduction in caries experience in 2013 when compared with 1995 (5 municipalities showed less than 10% reduction). Only 15 (5.5%) municipalities showed an increase in caries experience across the years.

**Fig. 2:**
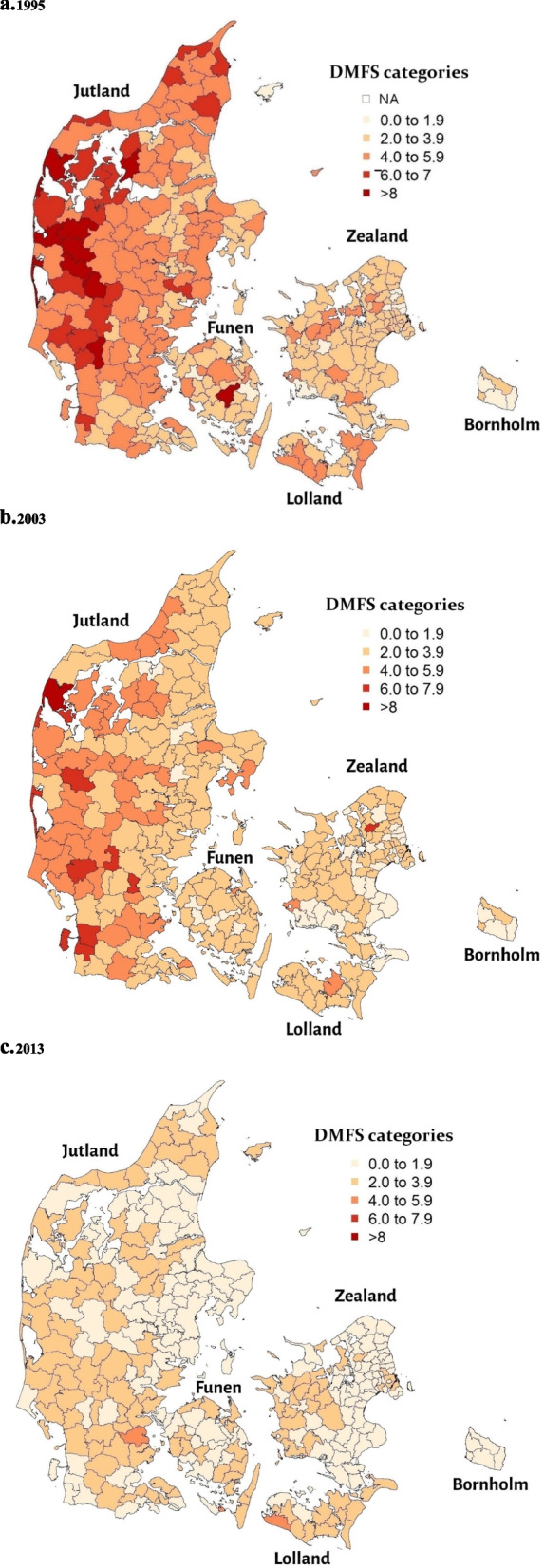
Geo-mapping of the magnitude of dental caries experience in the Danish municipalities based on observed mean DMFS values in (**a**) 1995, (**b**) 2003, and (**c**) 2013. Dental caries was measured by the decayed, missing, and filled tooth surface (DMFS) index. The observed mean DMFS values in the Danish municipalities were categorised into 5 groups: 0.0–1.9, 2.0–3.9, 4.0–5.9, 6.0–7.9, ≥ 8. Abbreviation: NA = not available (raw DMFS data not available for two municipalities, namely, Sakskøbing and Møldrup)

Despite the general decline in caries experience in the municipalities, geographic inequalities in caries experience among the municipalities were noted in all 3 years; the inequalities were smaller in 2013 than in 1995 and 2003 (Fig. 2: Geo-mapping of the magnitude of dental caries experience in the Danish municipalities based on observed mean DMFS values in (a) 1995, (b) 2003, and (c) 2013). In all three years, municipalities with higher caries experience were concentrated in Western, Northern, and Southern Jutland, Western Zealand, Lolland, and Eastern and Southern Funen (Fig. 2).

Adjusted mean DMFS ratios and the corresponding 95% CIs for the individual-level SEP variables from Model 1 are shown in Table 1. Increasing relative socioeconomic inequalities in caries were seen with all SEP indicators with significant graded associations in all three years. For example, in 2013, being from a lower parental education subgroup was associated with up to 2.31-fold (95% CI: 2.18–2.46) higher caries experience compared to being from the highest parental education subgroup (Table 1). Significant SEP–caries associations persisted in the joint analysis (Model 2) even after incorporating the fixed effect of study year. For example, having parents with basic education corresponded to 1.91-fold (95% CI: 1.86–1.95) higher caries experience compared with having parents with high education (Table 1).

**Table 1:** Caries ratio estimates (with 95% credible intervals) for the various parental socioeconomic position subgroups in the three study years (1995, 2003, and 2013) and in the overall pooled population of 15-year-olds. Caries experience was estimated using the decayed (D), missing (M), and filled (F) tooth surfaces (DMFS) index

Parental Socioeconomic Groups	1995^a^	2003^a^	2013^a^	Overall^b^
	*N* = 48,900	*N* = 50,195	*N* = 50,713	*N* = 149,808
**Parental education**				
Basic (Up to 10 years)	1.77 (1.71–1.83)^c^	1.81 (1.74–1.88)^c^	2.31 (2.18–2.46)^c^	1.91 (1.86–1.95)^c^
Medium (11–12 years)	1.42 (1.38–1.46)^c^	1.35 (1.31–1.39)^c^	1.49 (1.44–1.54)^c^	1.43 (1.41–1.46)^c^
High (13 or more years)	1.00 (Ref)	1.00 (Ref)	1.00 (Ref)	1.00 (Ref)
**Model parameters**				
Zero-probability parameter for ZINB	0.08 (0.07–0.10)^c^	0.11 (0.09–0.12)^c^	0.06 (0.04–0.08)^c^	0.05 (0.05–0.07)^c^
% of total variation due to spatial correlation	15.9	3.8	31.9	10.0
**Parental occupational social class**				
Out of labour market	1.88 (1.79–1.98)^c^	1.74 (1.65–1.84)^c^	2.11 (1.97–2.25)^c^	1.94 (1.88–2.01)^c^
Basic skill level	1.46 (1.40–1.52)^c^	1.43 (1.36–1.50)^c^	1.44 (1.36–1.53)^c^	1.45 (1.41–1.49)^c^
Intermediate skill level	1.17 (1.12–1.23)^c^	1.05 (0.99–1.11)	1.02 (0.96–1.10)	1.09 (1.06–1.13)^c^
Highest skill level	1.00 (Ref)	1.00 (Ref)	1.00 (Ref)	1.00 (Ref)
**Model parameters**				
Zero-probability parameter for ZINB	0.09 (0.07–0.12)^c^	0.11 (0.09–0.12)^c^	0.07 (0.05–0.10)^c^	0.06 (0.05–0.07)^c^
% of total variation due to spatial correlation	16.9	4.5	32.8	11.5
**Equivalised disposable household income**				
1st quartile (< 25%)	1.43 (1.38–1.49)^c^	1.58 (1.51–1.64)^c^	2.14 (2.02–2.26)^c^	1.69 (1.65–1.73)^c^
2nd quartile (25%–50%)	1.27 (1.23–1.32)^c^	1.37 (1.32–1.42)^c^	1.46 (1.39–1.54)^c^	1.37 (1.34–1.40)^c^
3rd quartile (50%–75%)	1.15 (1.11–1.18)^c^	1.15 (1.11–1.19)^c^	1.14 (1.08–1.19)^c^	1.15 (1.12–1.17)^c^
4th quartile (> 75%)	1.00 (Ref)	1.00 (Ref)	1.00 (Ref)	1.00 (Ref)
**Model parameters**				
Zero-probability parameter for ZINB	0.08 (0.07–0.09)^c^	0.10 (0.09–0.12)^c^	0.07 (0.04–0.10)^c^	0.05 (0.04–0.06)^c^
% of total variation due to spatial correlation	18.0	5.2	34.9	10.7

*Abbreviation*: *ZINB* zero-inflated negative binomial model

^a^Full spatial model adjusted for individual-level (country of origin, immigration status, household type, and number of persons in the family) and neighbourhood-level variables (Gini index; proportion of individuals within municipalities who were unemployed, had low education, were unmarried/non-cohabitating; proportion of household overcrowding, non-home ownership, and single parent households within municipalities)

^b^Full spatiotemporal model adjusted for assessment year in addition to the individual- and neighbourhood-level variables

^c^Significant result

The geographically structured random effect accounted for 15.9%–18.0%, 3.8%–5.2%, and 31.9%–34.9% of the residual variability in 1995, 2003, and 2013, respectively (Table 1). This indicates some degree of spatial autocorrelation of DMFS scores in the municipalities, implying that near neighbouring municipalities are somewhat correlated in terms of caries experience. Meanwhile, 65.1%–96.2% of the total residual variance in the study years was geographically unstructured.

The sensitivity analysis varying the prior specifications of the random effects showed that changing the parameter values of the log-gamma distributions (Model 3) did not alter the parameter estimates.

## Discussion

This large-scale investigation revealed a decrease in the magnitude of caries experience in almost all socioeconomic groups and Danish municipalities over the 18-year study period. However, geographic inequalities in caries persisted over the years, with higher caries levels being largely concentrated in a few municipalities in areas of Denmark that are generally considered as being comparatively deprived [24]. Relative socioeconomic inequalities also persisted over the years with marked social gradients in caries experience; consequently, the lower an adolescent’s parental SEP, the higher his/her caries levels were likely to be. The significant graded associations between the individual-level SEP variables and caries persisted over time even after adjustment for a range of individual- and neighbourhood-level confounders and the effect of assessment year.

### Strengths and limitations

The major strengths of this study are the large nationwide population and inclusion of all Danish municipalities, enhancing their representativeness of the Danish adolescent population. The use of national register data of proven validity decreases the scope for misclassification of exposures and outcomes and limits biases such as recall and non-response bias [25]. Another strength of the study is that all three commonly used individual-level SEP variables, namely, education, occupational social class, and income, were examined in the study. For a multidimensional construct like SEP, any single SEP indicator is unlikely to encompass the entirety of the effect of SEP on caries. Analysing multiple indicators helps minimise residual confounding through unmeasured socioeconomic circumstances [26]. Furthermore, in contrast to most of the earlier studies in this area (e.g., Ekstrand et al. 2010 [6], Nørrisgaard et al. 2016 [7]), advanced regression modelling was implemented that allowed simultaneous adjustment for several potential individual- and neighbourhood-level confounders as well as control for geographic clustering. This would potentially reduce residual confounding further and improve the precision of the estimates.

Potential limitations of this study include the possibility of selection bias despite the large nationwide population. This is because adolescents lacking dental data and data on the SEP indicators and residence municipality were excluded. Approximately 15% to 23% of the 15-year-olds in the three study years were excluded because they were not registered in SCOR. This could engender selection bias if being registered in SCOR, i.e., if undergoing a (mandatory) dental examination at age 15, depended on both the exposure (SEP) and the outcome (caries) [27]. The possibility that the exposure (SEP) determines whether or not an adolescent undergoes a dental examination is mitigated by the fact that when the included (i.e., had a dental examination) and excluded (no dental examination) 15-year-olds were compared, no marked differences were seen in terms of the proportions of individuals comprising each SEP subgroup (Appendix 1 a. 1995; b. 2003; c. 2013). Prior reports imply greater utilisation of dental services by children and adolescents from lower SEP backgrounds in the Nordic countries (presumably because of having higher disease levels and [universal] access to dental care) [2, 28]. Thus, it is less likely that diseased individuals from lower SEP backgrounds are not being able to access care and are systematically missing from SCOR. A possibility exists that the lack of a dental examination at age 15 may be due to relocation issues; however, these are unlikely to be systematically related to the study variables. It may also be that the adolescents had extended recall intervals (i.e., less frequent dental examinations) because of low risk of caries or other dental problems [29]. Absence of individuals without caries may reduce the representativeness of the study population. However, as indicated earlier, in terms of the study exposures and covariates, these individuals were not systematically different from those having dental caries data in SCOR. Therefore, because of its inherent property of scale invariance [30], the relative caries ratio estimates from this study would not change even if these individuals without disease were included. Thus, missing observations are not likely to induce significant selection bias in this study.

Misclassification of caries is a possibility in this study because many different uncalibrated dental examiners from all over Denmark perform diagnosis and recording of caries in SCOR. To alleviate misclassification, we limited caries diagnosis to cavitated lesions only (which are more clearly visible to the naked eye). Moreover, the risks of misclassification is lowered because the caries recording system in SCOR follows a relatively simple coding methodology based on detailed guidelines provided by the Danish Health Authority [31]. In addition, the diagnostic criteria for caries in Denmark are well defined and correspond with WHO standards [9]. Despite this, independent errors in caries diagnosis or data entry may occur. The impact of such errors depends on whether they are dependent and/or differential. In the Danish context, any such individual error would be independent and non-differential with respect to the exposure variables, as these variables were recorded autonomously in the registers [2]. Thus, misclassification is unlikely to be an important weakness in this study.

To minimise individual- and neighbourhood-level confounding, ethnicity, household size and type, and diverse factors related to neighbourhood income and deprivation were taken into consideration. Moreover, several important background factors were held constant for the study population in each assessment year, including universal access to dental and medical care and a basic welfare system. Nevertheless, residual confounding is still possible because of unmeasured potential common causes of the SEP exposures and the outcome (caries). These could be related to the family environment (e.g., parental somatic and mental disorders, family functioning etc.) as well as factors outside of the family environment. At the neighbourhood level, these could likely include contextual elements specific to municipalities (e.g., infrastructural aspects of dental care management such as children:dentist and auxiliary personnel:dentist ratios, the proximity of homes and schools to clinics, transportation systems affecting mobility and impetus to seek dental care, the quality of food available in stores and barriers to making healthier food choices, the degree of social support and social cohesion) [32, 33]. Owing to the complexity of measuring neighbourhood characteristics, such contextual aspects of the neighbourhood framework were likely not adequately taken into account by the variables included in study.

In terms of defining the neighbourhood framework per se, we used pre-2007 municipalities (*n* = 275), which provided a finer geographical resolution than the current set of municipalities (*n* = 98). We chose municipalities as geographical units because of their fundamental role in the organisation and implementation of dental care in Denmark. Moreover, data on neighbourhood income and deprivation, including the Gini index, are available for municipalities. However, it may be argued that some municipalities are relatively large and heterogeneous areas with considerable within-municipality variations in individual-level socioeconomic factors and caries levels (e.g., Copenhagen). A smaller, more homogenous unit might be more reflective of an individual’s immediate neighbourhood. Alternative definitions of neighbourhoods relevant to oral health may be based on locations of services such as dental care institutions or proximity to stores selling sugary foods.

#### Interpretation of the results

The aforementioned considerations on strengths and limitations along with the use of large-scale nationwide data suggest that our study results may be generalisable to at least the Danish and possibly the Scandinavian adolescent populations. The results signify that the existing tax-financed universal care provisions, while contributing to an overall reduction in caries experience among adolescents, have not alleviated geographic and socioeconomic inequalities in caries over time. Studies in various locations around the world, including Sweden [8], Scotland [34], Australia [35], and USA [32], have revealed geographic inequalities in caries. In regional studies within Denmark, geographical variation in caries experience among Danish adolescents (*n* = 1509) [7] and adults (*n* = 1115) [36] have also been reported previously. In this nationwide study, the persistence of inequalities in caries over two decades along with spatial autocorrelation imply that potential neighbourhood-level caries determinants that transcend administrative (municipal) boundaries likely play a role in the geographic patterning of caries. These could include contextual factors such as shared policies, compositional characteristics such as shared socioeconomic factors or behavioural and cultural practices, and environmental factors such as fluoride concentration in public water supplies. Further investigative effort is required to identify and assess the impact of these factors in causing clustering of caries among neighbouring municipalities. Moreover, a broader understanding of the processes through which neighbourhoods can affect dental caries may be gained by assessing contextual elements of neighbourhoods, over and above compositional elements. These could comprise aspects of the dental care management infrastructure; proximity of homes and schools to clinics, supermarkets, and fast-food chains; and degree of social support and social cohesion.

The socioeconomic gradients in caries experience observed in this study resembles the social group differentials in caries experience observed in previous studies, including studies from countries lacking an organised universal public dental care system like Denmark [37–40]. In general, accounting for neighbourhood-level measures is considered to flatten socioeconomic gradients and attenuate the effect of individual-level factors on health outcomes [41]. However, the SEP–caries associations in this study were similar to those observed in an earlier mono-level study based on nearly the same Danish adolescent population [2]. This unequivocally highlights the importance of SEP as a key determinant of dental caries and caries inequalities among adolescents in Denmark.

Overall, however, the fact that caries experience among adolescents in Denmark has improved over the last two decades across the board is undeniable. Reducing the persisting geographic and socioeconomic inequalities in caries, and indeed other non-communicable diseases sharing risk factors (e.g., obesity, diabetes), could be seen as the holy grail of the public health effort in Denmark in the future. This study provides a pathway for such effort by identifying key socioeconomic determinants and groups as well as geographic patterns and areas for which interventions and resources may best be proportionally targeted. Based on the study results, from a clinical perspective, it would be prudent to accord additional resources and supportive and preventive measures for adolescents from lower SEP backgrounds and/or residing in the relatively deprived municipalities experiencing higher caries levels. This should of course be accompanied by efforts to improve the socioeconomic conditions and opportunities among those worse off.

## Conclusion

Between 1995 and 2013, overall caries experience among adolescents in Denmark reduced. Such decline was witnessed in almost all socioeconomic subgroups and Danish municipalities. Nevertheless, geographic inequalities in caries persisted over the years with higher caries levels being largely concentrated in the relatively poorer areas of Denmark. Relative socioeconomic inequalities in caries also persisted with robust graded associations between parental SEP and dental caries observed even after adjustment for an array of individual and neighbourhood-level factors. Reducing these inequalities would require concerted focus on adolescents from lower SEP backgrounds and/or residing in the relatively deprived municipalities in Denmark with higher caries experience.

### Supplementary Information

**Additional file 1: Appendix 1.** Comparison of 15-year-olds with and without dental data according to the different sociodemographic categories^†^

## Data Availability

The data that support the findings of this study are available from the Danish Health Data Authority and Statistics Denmark; however, restrictions apply to the availability of these data, which were used under license for the current study. Thus, the data are not publicly available. Statistics Denmark may be contacted for queries regarding the data. Statistics Denmark. Sejrøgade 11, 2100 Copenhagen, Denmark. Email: dst@dst.dk. Ph: + 45 39 17 39 17.

## Abbreviations

BYM: Besag-York-Mollié statistical model
CAR: Conditionally autoregressive
CI: Credible interval
CRS: (Danish) Civil Registration System
DMFS: Decayed (D), missing (M), and filled (F) tooth surfaces index
INLA: Integrated nested Laplace approximations
OECD: Organisation for Economic Co-operation and Development
PIN: Personal identification number
SCOR: Sundhedsstyrelsens Centrale Odontologiske Register (Danish national dental database)
SEP: Socioeconomic position
WHO: World Health Organization

## References

[1] Wen PYF, Chen MX, Zhong YJ, Dong QQ, Wong HM (2022). Global burden and inequality of dental caries, 1990 to 2019. J Dent Res.

[2] Sengupta K, Christensen LB, Mortensen LH, Skovgaard LT, Andersen I (2017). Trends in socioeconomic inequalities in oral health among 15-year-old Danish adolescents during 1995–2013: a nationwide, register-based, repeated cross-sectional study. Community Dent Oral Epidemiol.

[3] Sengupta K, Ersbøll AK, Christensen LB, Mortensen LH, Andersen I (2020). Inequality, familial aggregation, and risk prediction of caries in siblings. JDR Clin Trans Res.

[4] Herke M, Moor I, Winter K, Hoffmann S, Spallek J, Hilger-Kolb J, Pischke C, Dragano N, Novelli A, Richter M (2020). Role of contextual and compositional characteristics of schools for health inequalities in childhood and adolescence: protocol for a scoping review. BMJ Open.

[5] Arcaya MC, Arcaya AL, Subramanian SV (2015). Inequalities in health: definitions, concepts, and theories. Glob Health Action.

[6] Ekstrand KR, Christiansen ME, Qvist V, Ismail A (2010). Factors associated with inter-municipality differences in dental caries experience among Danish adolescents. An ecological study. Community Dent Oral Epidemiol..

[7] Nørrisgaard PE, Qvist V, Ekstrand K (2016). Prevalence, risk surfaces and inter-municipality variations in caries experience in Danish children and adolescents in 2012. Acta Odontol Scand.

[8] Strömberg U, Magnusson K, Holmén A, Twetman S (2011). Geo-mapping of caries risk in children and adolescents - a novel approach for allocation of preventive care. BMC Oral Health.

[9] World Health Organisation (WHO) (2013). Oral health surveys - basic methods.

[10] Thygesen LC, Ersbøll AK (2011). Danish population-based registers for public health and health-related welfare research: introduction to the supplement. Scand J Public Health.

[11] Jensen VM, Rasmussen AW (2011). Danish education registers. Scand J Public Health.

[12] Baadsgaard M, Quitzau J (2011). Danish registers on personal income and transfer payments. Scand J Public Health.

[13] Petersson F, Baadsgaard M, Thygesen LC (2011). Danish registers on personal labour market affiliation. Scand J Public Health.

[14] Registry and variable summaries. Statistics Denmark. 2019. https://www.dst.dk/extranet/ForskningVariabellister/FAIN%20-%20Husstande%20og%20familier.html. Accessed 9 Apr 2022.

[15] Pedersen CB (2011). The Danish civil registration system. Scand J Public Health.

[16] Statbank. Statistics Denmark. 2021. https://www.statbank.dk/statbank5a/default.asp?w=1280. Accessed 9 Apr 2022.

[17] United Nations Educational, Scientific and Cultural Organisation (UNESCO). International Standard Classification of Education - ISCED 2011. UNESCO Institute for Statistics. 2011. http://uis.unesco.org/sites/default/files/documents/international-standard-classification-of-education-isced-2011-en.pdf. Accessed 9 Apr 2022.

[18] FAMAEKVIVADISP (equivalised disposable income for the family). Statistics Denmark. 2021. https://www.dst.dk/da/Statistik/dokumentation/Times/familieindkomst/famaekvivadisp. Accessed 9 Apr 2022.

[19] Besag J, York J, Mollie A (1991). Bayesian image restoration, with application in spatial statistics with discussion. Ann Inst Stat Math.

[20] Miller HJ (2004). Tobler’s first law and spatial analysis. Ann Assoc Am Geogr.

[21] Blangiardo M, Cameletti M, Baio G, Rue H (2013). Spatial and spatio-temporal models with R-INLA. Spat Spatiotemporal Epidemiol.

[22] Rue H, Martino S, Chopin N. R-INLA package. 2021. http://www.r-inla.org/. Accessed 9 Apr 2022.

[23] Danish Health Authority (2018). The municipal dental care (Den kommunale tandpleje).

[24] Ministry of Economic and Business Affairs. Denmark in balance in a global world (Danmark i balance i en global verden). Albertslund, Denmark; 2010. https://www.regeringen.dk/aktuelt/tidligere-publikationer/danmark-i-balance-i-en-global-verden/ (in Danish). Accessed 9 Apr 2022.

[25] Thygesen LC, Ersbøll AK (2014). When the entire population is the sample: strengths and limitations in register-based epidemiology. Eur J Epidemiol.

[26] Oakes M, Andrade K, Oakes M, Kaufman J (2017). The measurement of socioeconomic status. Methods in social epidemiology.

[27] Hernán MA, Robins JM, Hernán MA, Robins JM (2020). Selection bias. Causal inference: what if.

[28] Virtanen JI, Berntsson LT, Lahelma E, Köhler L, Murtomaa H (2007). Children’s use of dental services in the five Nordic countries. J Epidemiol Community Health.

[29] Strömberg U, Holmn A, Magnusson K, Twetman S (2012). Geo-mapping of time trends in childhood caries risk–a method for assessment of preventive care. BMC Oral Health.

[30] Erreygers G, Van Ourti T (2011). Measuring socioeconomic inequality in health, health care and health financing by means of rank-dependent indices: a recipe for good practice. J Health Econ.

[31] Helm S (1973). Recording system for the Danish child dental health services. Community Dent Oral Epidemiol.

[32] Tellez M, Sohn W, Burt BA, Ismail AI (2006). Assessment of the relationship between neighborhood characteristics and dental caries severity among low-income African-Americans: a multilevel approach. J Public Health Dent.

[33] Ekstrand KR, Christiansen ME, Qvist V (2003). Influence of different variables on the inter-municipality variation in caries experience in Danish adolescents. Caries Res.

[34] Levin KA, Davies CA, Douglas GV, Pitts NB (2010). Urban-rural differences in dental caries of 5-year-old children in Scotland. Soc Sci Med.

[35] Brennan DS, Spencer AJ (2004). Changes in caries experience among Australian public dental patients between 1995/96 and 2001/02. Aust N Z J Public Health.

[36] Krustrup U, Petersen PE (2007). Dental caries prevalence among adults in Denmark–the impact of socio-demographic factors and use of oral health services. Community Dent Health.

[37] Funieru C, Twetman S, Funieru E, Dumitrache AM, Sfeatcu RI, Baicus C (2014). Caries experience in schoolchildren in Bucharest, Romania: the PAROGIM study. J Public Health Dent.

[38] Mathur MR, Tsakos G, Millett C, Arora M, Watt R (2014). Socioeconomic inequalities in dental caries and their determinants in adolescents in New Delhi, India. BMJ Open.

[39] Perera I, Ekanayake L (2008). Social gradient in dental caries among adolescents in Sri Lanka. Caries Res.

[40] Slade GD, Sanders AE (2018). Two decades of persisting income-disparities in dental caries among U.S. children and adolescents. J Public Health Dent.

[41] Pampalon R, Hamel D, Gamache P (2009). A comparison of individual and area-based socio-economic data for monitoring social inequalities in health. Health Rep.

