# Supplementary material for: Development of geographic inequality in dental caries and its association with socioeconomic factors over an 18-year period in Denmark
Source: BMC Oral Health. 2023 Sep 14;23:662. doi: 10.1186/s12903-023-03373-5 (PMC10500719; doi:10.1186/s12903-023-03373-5)
Supplement: Supplementary file 1 — Additional file 1: Appendix 1. Comparison of 15-year-olds with and without dental data according to the different sociodemographic categories† [file 12903_2023_3373_MOESM1_ESM.docx]

# Supplementary material.

**Appendix 1. Comparison of 15-year-olds with and without dental data according to the different sociodemographic categories^†^**

**a. 1995**

| **Sociodemographic groups (1995)** | **Dental data for the 15-year-olds** | | | |
| --- | --- | --- | --- | --- |
|  | **Yes** | | **No** | |
|  | **N** | **%** | **Frequency** | **Percent** |
| **Gender** |  |  |  |  |
| Male | 24,846 | 50.81 | 4272 | 51.7 |
| Female | 24,054 | 49.19 | 3991 | 48.3 |
| **Ethnicity/immigrant status** |  |  |  |  |
| Danes | 46,740 | 95.58 | 7790 | 94.3 |
| Immigrants | 1200 | 2.45 | 279 | 3.4 |
| Descendants of immigrants | 960 | 1.96 | 194 | 2.4 |
| **Country of origin** |  |  |  |  |
| Denmark and Western countries | 46,922 | 95.96 | 7831 | 94.8 |
| Non-Western countries | 1978 | 4.04 | 432 | 5.2 |
|  |  |  |  |  |
| **No. of children in the family** |  |  |  |  |
| 1 child | 10,827 | 22.14 | 1898 | 23.0 |
| 2 children | 24,650 | 50.41 | 3988 | 48.3 |
| 3 children | 9500 | 19.43 | 1551 | 18.8 |
| 4 or more children | 3923 | 8.02 | 826 | 10.0 |
|  |  |  |  |  |
| **No. of persons in the family** |  |  |  |  |
| 1–2 persons | 4349 | 8.89 | 852 | 10.3 |
| 3–5 persons | 41,712 | 85.3 | 6835 | 82.7 |
| >5 persons | 2839 | 5.81 | 576 | 7.0 |
| **Household type** |  |  |  |  |
| Traditional family (living with both parents) | 33,644 | 68.8 | 5502 | 66.6 |
| Single-parent family (living with one parent) | 8402 | 17.18 | 1566 | 19.0 |
| Reconstructed family (parent with a new partner) | 5994 | 12.26 | 997 | 12.1 |
| Children not living with parents | 860 | 1.76 | 198 | 2.4 |
| **Highest parental education** |  |  |  |  |
| Basic (Up to 10 years) | 9430 | 19.28 | 1775 | 21.5 |
| Medium (11–12 years) | 25,067 | 51.26 | 4067 | 49.2 |
| High (13 or more years) | 14,403 | 29.45 | 2421 | 29.3 |
| **Highest parental occupational social class** | |  |  |  |
| Highest skill level | 5005 | 10.24 | 853 | 10.3 |
| Intermediate skill level | 9451 | 19.33 | 1646 | 19.9 |
| Basic skill level and students | 28,587 | 58.46 | 4487 | 54.3 |
| Out of labour market | 5857 | 11.98 | 1277 | 15.5 |

**b. 2003**

| **Sociodemographic Groups (2003)** | **Dental data for the 15-year-olds** | | | |
| --- | --- | --- | --- | --- |
|  | **Yes** | | **No** | |
|  | **Frequency** | **Percent** | **Frequency** | **Percent** |
| **Gender** |  |  |  |  |
| Male | 25,555 | 51.37 | 5240 | 52.1 |
| Female | 24,194 | 48.63 | 4812 | 47.9 |
| **Immigrant status** |  |  |  |  |
| Danes | 45,537 | 91.53 | 9161 | 91.1 |
| Immigrants | 2358 | 4.74 | 500 | 5.0 |
| Descendants of immigrants | 1854 | 3.73 | 391 | 3.9 |
| **Country of origin** |  |  |  |  |
| Denmark and Western countries | 45,843 | 92.15 | 9231 | 91.8 |
| Non-Western countries | 3906 | 7.85 | 821 | 8.2 |
|  |  |  |  |  |
| **No. of children in the family** |  |  |  |  |
| 1 child | 9308 | 18.71 | 2019 | 20.1 |
| 2 children | 23,700 | 47.64 | 4662 | 46.4 |
| 3 children | 11,631 | 23.38 | 2246 | 22.3 |
| 4 or more children | 5110 | 10.27 | 1125 | 11.2 |
| **No. of persons in the family** |  |  |  |  |
| 1–2 persons | 4107 | 8.26 | 973 | 9.7 |
| 3–5 persons | 41,660 | 83.74 | 8266 | 82.2 |
| >5 persons | 3982 | 8.0 | 813 | 8.1 |
| **Household type** |  |  |  |  |
| Traditional family (living with both parents) | 33,324 | 66.98 | 6427 | 64.0 |
| Single-parent family (living with one parent) | 9198 | 18.49 | 2082 | 20.7 |
| Reconstructed family (parent with a new partner) | 6459 | 12.98 | 1328 | 13.2 |
| Children not living with parents | 768 | 1.54 | 215 | 2.1 |
| **Highest parental education** |  |  |  |  |
| Basic (Up to 10 years) | 6862 | 13.79 | 1514 | 15.1 |
| Medium (11–12 years) | 26,386 | 53.04 | 5226 | 52.0 |
| High (13 or more years) | 16,501 | 33.17 | 3312 | 33.0 |
| **Highest parental occupational social class** | |  |  |  |
| Highest skill level | 3890 | 7.82 | 764 | 7.6 |
| Intermediate skill level | 6684 | 13.44 | 1314 | 13.1 |
| Basic skill level and students | 26,940 | 54.15 | 5128 | 51.0 |
| Out of labour market | 12,235 | 24.59 | 2846 | 28.3 |

**c. 2013**

| **Sociodemographic Groups (2013)** | **Dental data for the 15-year-olds** | | | |
| --- | --- | --- | --- | --- |
|  | **Yes** | | **No** | |
|  | **Frequency** | **Percent** | **Frequency** | **Percent** |
| **Gender** |  |  |  |  |
| Male | 26,037 | 51.34 | 7678 | 51.4 |
| Female | 24,676 | 48.66 | 7267 | 48.6 |
| **Immigrant status** |  |  |  |  |
| Danes | 45,928 | 90.56 | 13,627 | 91.2 |
| Immigrants | 1035 | 2.04 | 290 | 2.0 |
| Descendants of immigrants | 3750 | 7.4 | 1028 | 6.9 |
| **Country of origin** |  |  |  |  |
| Denmark and Western countries | 46,230 | 91.16 | 13,726 | 91.8 |
| Non-Western countries | 4483 | 8.84 | 1219 | 8.2 |
|  |  |  |  |  |
| **No. of children in the family** | |  |  |  |
| 1 child | 8768 | 17.29 | 2733 | 18.3 |
| 2 children | 24,727 | 48.76 | 7374 | 49.3 |
| 3 children | 12,375 | 24.4 | 3497 | 23.4 |
| 4 or more children | 4843 | 9.55 | 1341 | 9.0 |
|  |  |  |  |  |
| **No. of persons in the family** |  |  |  |  |
| 1–2 persons | 4687 | 9.24 | 1463 | 9.8 |
| 3–5 persons | 42,510 | 83.82 | 12,490 | 83.6 |
| >5 persons | 3516 | 6.93 | 992 | 6.6 |
| **Household type** |  |  |  |  |
| Traditional family (living with both parents) | 31,774 | 62.65 | 9287 | 62.1 |
| Single-parent family (living with one parent) | 11,557 | 22.79 | 3556 | 23.8 |
| Reconstructed family (parent with a new partner) | 6517 | 12.85 | 1873 | 12.5 |
| Children not living with parents | 865 | 1.71 | 229 | 1.5 |
| **Highest parental education** |  |  |  |  |
| Basic (Up to 10 years) | 4333 | 8.54 | 1198 | 8.0 |
| Medium (11–12 years) | 26,149 | 51.56 | 7488 | 50.1 |
| High (13 or more years) | 20,231 | 39.89 | 6259 | 41.9 |
| **Highest parental occupational social class** | | |  |  |
| Highest skill level | 5012 | 9.88 | 1575 | 10.5 |
| Intermediate skill level | 8120 | 16.01 | 2457 | 16.4 |
| Basic skill level and students | 24,879 | 49.06 | 7121 | 47.7 |
| Out of labour market | 12,702 | 25.05 | 3792 | 25.4 |

^†^Excluded are those having missing data pertaining to one or more study variables (including those with a negative household income)
